# The creative behavior of virtual idol fans: a psychological perspective based on MOA theory

**DOI:** 10.3389/fpsyg.2023.1290790

**Published:** 2023-11-21

**Authors:** Qingnian Wang, Shuyu Long, Yanfei Zeng, Lan Tang, Yunpei Wang

**Affiliations:** ^1^School of Journalism and Communication, South China University of Technology, Guangzhou, China; ^2^School of International Education, South China University of Technology, Guangzhou, China; ^3^School of Economics and Management, South China Normal University, Guangzhou, China

**Keywords:** creative behavior, virtual idols, fans, MOA theory, psychological perspective

## Abstract

Breakthroughs in digital technology are accelerating the development and commercialization of virtual idols. They are overturning the traditional one-way communication between idols and fans, turning fans into producers and consumers. Therefore, identifying the motivations for fan creation can regulate and guide the creative behavior of fans and turn their creativity into productivity. This paper took “the factors influencing fans’ spontaneous participation in creating virtual idols” as the primary research question, took the Motivation Opportunity Ability (MOA) theory as the research framework, used questionnaires as the primary research method, and combined the relevant research on motivation theory and self-determination theory to explore fans’ creative behavior from the psychological perspective in depth. The empirical tests revealed that in the motivation dimension, interest motivation, achievement motivation, social motivation, and utility motivation all positively influenced fans’ creative behavior; in the opportunity dimension, fans’ perceived cost had a significant negative moderating effect on the influence of interest motivation, social motivation, and utility motivation on fans’ creative behavior; fans’ perceived community atmosphere was substantial. The moderating effect of knowledge and skills on the capability dimension was insignificant. For the virtual idol industry, the future development of the industry could not only focus on IP image creation and content production but also effectively stimulate fans’ creative motivation through the creation of an excellent community atmosphere, the provision of targeted creator services, and the reduction of fans’ perceived costs in the creation process.

## 1 Introduction

Under the wave of the digital economy, virtual idols are becoming one of the most imaginative industries. According to the “2022 Research Report on the Development of China’s Virtual Human Industry” released by iMedia Research (2022), the number of virtual human-related enterprises has doubled since 2021. The overall market size and core market size driven by virtual idols were 107.49 billion RMB and 6.22 billion RMB, respectively. The growth rate of their market size has exceeded 50% for four consecutive years, and it was expected to reach 640.27 billion RMB and 48.06 billion RMB, respectively, by 2025. The market showed a penetration trend from online channels to offline spaces and from niche circles to mainstream cultural circles. Virtual idols are aerial images based on digital technology and even a “concept” jointly created by virtual idol production companies and fans. With the support of technology, virtual idols have strong plasticity, incredibly realistic forms, and natural expressions, which not only achieve breakthrough development but also provide more possibilities for fans to participate in creative activities. One of the essential characteristics of virtual idols is “sharing and co-creation and stimulating the creative power of fans is an integral part of the operation and management of virtual idols. Improving the ecology of creators has also become an essential part of the development of virtual idols. Therefore, from the perspective of fan creators, the psychological motivation and external influencing factors of fan participation in virtual idol creation are a new field worth exploring (Edmondson et al., 2004; Titrek and Celik, 2011; Dahling and Ruppel, 2016; Raza et al., 2017; UNCTAD, 2018; Ahmad et al., 2019; Peng et al., 2019; Choi et al., 2021; Malik et al., 2021; Wang et al., 2021; Cui et al., 2022; Schilling et al., 2022).

Virtual idol refers to a virtual character who is usually a singer, actor, idol, etc. It is artificially created using computer graphics like Lil Miquela, Imma, A-Soul, etc. In recent years, there have been many studies on fans’ involvement in the production and consumption of idols, but these have not yet been extended to the specific subject of virtual idol fans (Li and Chen, 2018; Zheng and Zhang, 2019; Choi et al., 2021; iMedia Research, 2022; Wang, 2022). However, most of the existing academic studies on fans of virtual idols stop at the cultural study of virtual idols and fans, neglecting to explore the factors influencing fans’ spontaneous participation in creation from the perspective of empirical research (Podsakoff et al., 2003; Shalley and Gilson, 2004; Shalley et al., 2009; Shin et al., 2019; Liu et al., 2023; Widodo et al., 2023). Virtual idol fans had significantly more initiative and creative power in the production and consumption of their idols, which made the innovative behavior of virtual idols more and more complex. Therefore, this paper aims to examine in depth the creative behavior of virtual idol fans from a psychological perspective phenomenon to better understand the nature of the virtual idol phenomenon (Wang et al., 2017; Yuan et al., 2019; Gelaidan et al., 2022; Cai et al., 2023; Liang et al., 2023).

The main contribution of this study lies in the following two aspects. In terms of the virtual idol industry, it not only helped to promote the development of virtual idol and fan creation but also provided suggestions and strategies for regulating and promoting fans’ creative behavior, ultimately achieving the aim of promoting the healthy and sustainable development of the virtual idol industry. In a theoretical sense, this paper used MOA theory to conduct research, combined self-determination theory and motivation theory to construct a research model of virtual idol fans’ creative behavior, and reasonably classified the factors influencing virtual idol fans’ creative behavior from three levels: motivation, ability, and opportunity, and finally conducts validation analysis through questionnaire survey method. This broadened the application area and research object of MOA theory to a certain extent (Anaza and Nowlin, 2017; Anwar, 2017; Aljawarneh and Atan, 2018; Liu et al., 2023; Lu et al., 2023).

The paper was structured into the following sections: firstly, a literature review of the relevant research areas; secondly, a description of the research design and the research methods used; a clarification of the findings of this study; and finally, an analysis of the findings and recommendations for the future marketing and management of virtual idols based on the findings. Overall, this paper applied MOA theory to the study of the factors influencing the creative behavior of virtual idol fans, provided an academic explanation for the spontaneous creative behavior of fans, and explored the factors influencing the creative behavior of virtual idol fans as well as the internal correlation between these factors. Based on the research findings, marketing management suggestions were provided for developing virtual idols, including suggestions for optimizing the design of creative incentives for virtual idol companies and online communities, as well as suggestions for the rational management and guidance of fans’ creative behavior.

## 2 Theoretical background and research hypotheses

### 2.1 Virtual idols

The rapid development of virtual idols has received widespread attention from scholars at home and abroad, focusing on four aspects: the study of the phenomenon of virtual idol development, critical reflection, business models, and fan culture.

The first was to analyze virtual idols at the phenomenological level and to give a general grasp of the new phenomenon of virtual idols. In his study of “celebrities,” Chris (2002) noted the fictionalized images of celebrities and called them “virtual celebrities,” whose image-building represented the tastes of the public at the time. Zhao (2017) explained the cultural attributes of virtual idols and unpacked them, arguing that they were a clever blend of technology, idol culture, and music culture, ultimately merging into a vibrant and emerging youth subculture. Most scholars agreed that virtual idols were avatars engaged in performing arts under the development of digital technology. They were “perfect images” constructed by the official and the fans, with fans’ at their core.

From a critical and reflective standpoint, studies from this perspective seek to explore the potential pitfalls of the rapid development of virtual idols and reveal the social issues involved. Existing studies have critically considered the development of virtual idols from the perspectives of gender hegemony (Black, 2012; Farrukh et al., 2023), subculture, post-human, semiotics, and consumer culture. Communication scholars mostly affirmed the cultural creativity brought by virtual idols but also remained wary of the possible alienation of cultural symbols, the dissolution of the boundaries between reality and imaginary, gender hegemony, and self-alienation brought about by technological development (He et al., 2020a,b; Liu et al., 2022; Wan et al., 2022).

Many studies explored the development of virtual idols from the perspective of business models. Guga (2015) believed that the success of virtual idols in business models was not only reflected in their progressive technology but also reflected in their characteristics as a combination of enterprise software products, pop stars, behavioral artists, and multimedia art products. Guo and Zhang (2022) analyzed the development of virtual idols and their marketing from the perspective of the fan economy, arguing that fan participation in the creation of virtual idols was the most important feature and advantage of virtual idols, which was conducive to enhancing the stickiness of fans and the commercialization value of idols. In general, the most incredible, most significant economic value and business model innovation of virtual idols was reflected in the openness of their creation technology and the unique fan co-creation operation model.

At present, related to the studies on the culture and operation model of virtual idols and fans, scholars mainly focused on cultural studies in communication, including theoretical perspectives such as mimetic social interaction (Liberman et al., 2001; Jha and Varkkey, 2018; Khalid et al., 2018; Zhao and Xia, 2019; Zhao and Liu, 2020; Zhang and Wang, 2021; Wang, 2022), participatory culture (Widmer et al., 2012; Wen, 2017; Men et al., 2018; Song, 2019; West et al., 2019), group identity (Zhan, 2019), identity construction and identity (Rhee and Choi, 2016; Li and Chen, 2018; Mitchell et al., 2019; Robbins and Judge, 2019; Zhang and Min, 2019; Nguyen et al., 2022), and emotional labor. From the perspective of mimetic social interaction, identity, the need for social security and the emotion of admiration were the ties that sustain the imitative social interaction between virtual idols and their fans (John, 2006; Zhang et al., 2015; Škerlavaj et al., 2018; Song et al., 2020; Tang et al., 2021). Most scholars noticed the transformation of virtual idol fans from consumers to producers in their analysis of virtual idols and fans and the resulting fan value.

### 2.2 The creative behavior of idol fans

The study of the creative behavior of fans first began with the subject of real-life icons. As early as the end of the 20th century, John Fiske referred to fans as “excessive readers,” arguing that they were not passive recipients, but were not only discerning but also productive, eager to create their own culture by “assembling” and “rewriting” the original texts (Postigo, 2007). With the advent of the Web 2.0 era, the connotations and forms of fan ‘participatory culture’ have been further expanded. Social media, video sites, and other UGC and PUC platforms have begun to enter the lives of the public, not only accelerating the formation of online communities linked by intriguing ties, but also widening the path of means of text production and circulation, providing fans with a platform to express their views and self-expression. Some studies also confirmed that fans did not just passively receive content, but also spontaneously evaluated it and created based on it (Zheng and Zhang, 2019). In the new media era, scholars have analyzed the drivers of fans’ participation in creative behavior, creative characteristics, and the construction and operation of fan creation communities from the perspectives of social psychology (Zhao and Hou, 2018), media economics, semiotics, and group identity and emotional payoff. In short, with the support of media technology, fans transformed from mere consumers to highly engaged producers and consumers, “productive consumers” who participated in original content, secondary re-creation, and even creative integration.

As a new thing, there were few studies on the creative behavior of virtual idol fans. Some studies analyzed the characteristics of virtual idol fans’ participation behavior and initially affirmed the high degree of autonomy of fans in the creation of virtual idols, as well as their cultural significance and commercial value, which also guided this study to explore the creative behavior of virtual idol fans (Yang and Peng, 2015; Shang et al., 2019; Park and Lee, 2021; Gelaidan et al., 2022; Wan et al., 2022; Widodo et al., 2023). Some scholars studied the creative motivations of virtual idol fans, mainly through descriptive analysis and qualitative research represented by in-depth interviews (Postigo, 2007; Fan and Zhang, 2015; Zhao and Hou, 2018; Zhan, 2019; Guo and Zhang, 2022; Zhang et al., 2022).

Overall, in the research on virtual idols, in-depth attention was paid to the problems faced by virtual idol fans in their creative practice, and few of them considered the individual motivation variables for fan creators without delving into the psychological motivation and environmental influencing factors of fan creative behavior. Although empirical studies focused on the motivations of fans’ creative behavior in the study of “real idols,” they did not extend to the emerging subject of virtual idols. Secondly, from the perspective of research methods, the research method for virtual idols was relatively homogeneous, lacking empirical examination of virtual idols and their fan creator groups, and failing to fully reflect the complete picture of fan creators. Finally, from the psychological perspective of the study, multiple factors influenced individuals’ spontaneous participation in creation, involving various influences such as personal and environmental factors. Based on this, this study focused on the factors influencing fans’ spontaneous participation in virtual idol creation. Quantitative research methods were used to explore the psychological motivation and environmental factors influencing fan creative behavior to help virtual idol companies more accurately understand the psychology and behavior of fan creators when shaping and maintaining virtual idol images.

### 2.3 Theoretical model

The MOA theoretical model is derived from studies on the motivation of individual information behavior. Based on earlier studies on motivation, MacInnis and Jaworski (1989) extracted and summarized reasonable factors in multiple relevant models and proposed a MOA theoretical model to explain the occurrence of individual behavior. MOA theory includes three pre-variables: Motive, Opportunity, and Ability. Its basic assumption is that motivation will directly impact behavior, and there is a complementary relationship between motivation, opportunity, and ability (Kim et al., 2022; Zhang et al., 2022). In other words, individual motivation will directly affect the final behavior, and ability and opportunity, playing a crucial regulatory role in this process.

MOA theoretical model is open and inclusive, and it does not have a specific list of variables, which provides an effective practical basic framework for analyzing the factors influencing individual behavior. Motivation refers to a kind of motivation that leads an individual to produce a specific behavior, generally expressed as willingness, interest, and the desire to process information. Opportunity is an abstract concept, mainly characterized by timeliness and advantage, which refers to the situation or external driving factors that have an impact on an individual’s implementation of a particular behavior (MacInnis and Jaworski, 1989; Binney et al., 2006; Eva et al., 2023). Specific behavior to the knowledge and skills needed for an individual to carry out a particular behavior or task. The relationship between the three factors in the MOA theoretical model can be understood as follows: motivation (M) is the precursor driving force of individual behavior and the direct factor of behavior occurrence; Opportunity (O) is the collection of many factors in the external environment that affect the production of behavior; Ability (A) is the skill that is necessary for the production of behavior. The combination of ability - opportunity - ability contributes to producing individual-specific behavior.

The MOA theoretical model was first used in the field of advertising and marketing and has now been widely used in the research of information-receiving behavior in the fields of communication and management, such as social marketing (Edmondson, 1999; Baer and Oldham, 2006; Maqbool et al., 2019; Chatterjee et al., 2021; Hwang et al., 2023), knowledge exchange, and knowledge sharing in virtual communities (Tierney and Farmer, 2011; Ghani et al., 2019; He et al., 2021; Wang et al., 2021; Chen et al., 2022), influencing factors of Internet knowledge payment behavior (Sijbom et al., 2017; Shang et al., 2019; Singh, 2019), and travelers’ intention to participate in social media (Yang et al., 2019; Ye et al., 2020; Yang et al., 2021), etc. Specifically on the level of users’ creative behavior, Fan and Zhang (2015) applied MOA theory to the study of users’ participation behavior in Q&A websites. They found that altruistic and reciprocal motives and opportunities provided by the platform, such as perceived ease of use, perceived economy, perceived website image, website atmosphere, as well as users’ ability in knowledge transformation and professional knowledge, which is an element related to user participation behavior (Briliana et al., 2015; Yang and Hu, 2019). Yang and Peng (2015) applied the MOA theory to research the driving mechanism of tourist knowledge sharing. They discovered that the three dimensions of motivation-opportunity-ability substantially impact users’ creative behavior. The main influencing factors are self-presentation, perceived entertainment, human-computer interaction, perceived interaction, and professional skills.

In general, the MOA theoretical model covers the individual factors and situational factors of behavior, and a large number of different studies have confirmed the applicability of the MOA theoretical model in other disciplines, it is an integrated analytical framework to explain the occurrence of individual behaviors.

**Figure 1:**
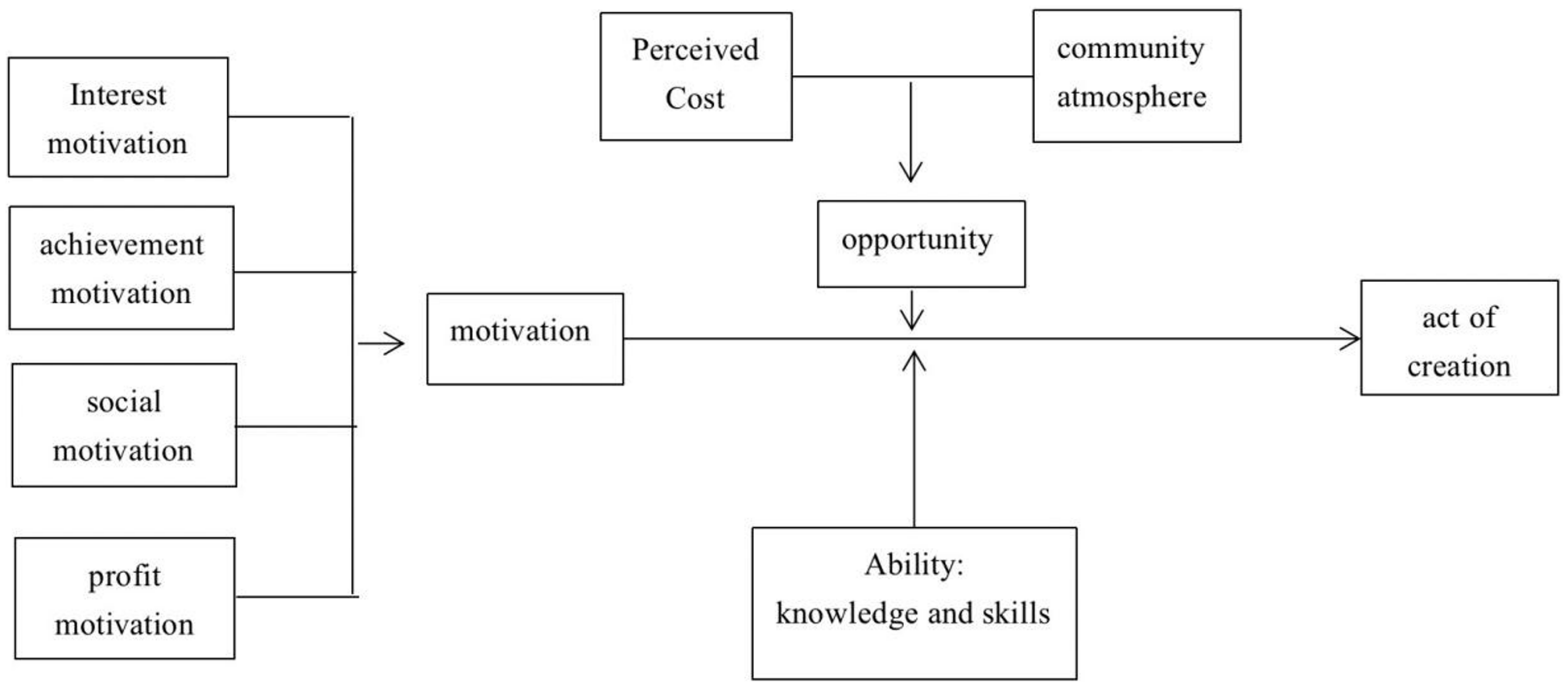
Model for studying the creative behavior of virtual idol fans from an MOA perspective.

This study took the MOA theoretical model as the theoretical framework, combined the research results of motivation theory and self-determination theory, and extracted the factors influencing fan creative behavior at the motivation, opportunity, and ability levels according to the characteristics of virtual idol creation and the characteristics of its fan groups. The self-determination theory holds that each individual has the basic psychological need for self-development, and divides this basic psychological need into autonomy, competence, and relatedness (Deci and Ryan, 2009). Based on the self-determination theory, the internal motivation of fans is divided into three dimensions: interest motivation, achievement motivation, and benefit motivation. Ryan and Deci (2000) pointed out that when individuals are intrinsically motivated, they will act out of intrinsic satisfaction, pleasure or challenge brought by activities, such as hunger, responsibility, altruism, and desire to be appreciated. Motivation theory divides fans’ creative motivation into internal motivation and external motivation. In the empirical research of fans’ creative behavior, motivation theory, and self-determination theory will provide more in-depth research for the research based on the MOA theoretical model. Specifically, the motivation level included four independent variables: interest motivation, achievement motivation, social motivation, and benefit motivation from the psychological perspective; the opportunity level included two moderating variables, perceived cost, and community atmosphere; and the ability level included the moderating variable of knowledge and skills. Finally, this paper constructed a research model that influenced the creative behavior of virtual idol fans (Figure 1).

### 2.4 Research hypotheses

This study drew on previous research, used the MOA theoretical model as the basic framework, and combined classical motivation theory research, self-determination theory, and interview data to propose the following research hypotheses.

Motivation refers to whether the individual’s subjective will to produce a particular behavior is satisfied, and it is the integration of multiple elements, such as will, interest, and expectation, in the determined behavior. Combined with the above studies on motivation theory and self-determination theory, four secondary variables affecting fans’ spontaneous participation in creating virtual idols are extracted from previous studies on fan groups’ participation behavior and user creation motivation. Therefore, hypothesis H1 is proposed.

*H1*: Creative motivation has a significant positive effect on the creative behavior of virtual idol fans. Four sub-variables were included: interest motivation, achievement motivation, social motivation, and benefit motivation.

Previous studies have shown that users’ content contribution behavior will be affected by perceived cost. For example, Fan and Zhang (2015) used the concept of “perceived economy” to represent the time and energy spent by users on the website platform and found that perceived economy will affect users’ contribution behavior in the dimension of “opportunity.” The creation of virtual idol fans is mostly a kind of “free labor,” and the fans pursue more non-material benefits in the creation, but the fans need to spend a lot of time and energy or even money into the creation. Therefore, only when the perceived cost of fan creators is small, they are more likely to participate in the creation. In addition, when individuals are engaged in community activities, they are often constrained by the community environment, and good relationship norms and atmosphere help fan creators to participate in the creation of virtual idols for a longer time. The community atmosphere can influence the fan creator’s perception of the feasibility of the behavior and the behavior that is likely to be rewarded, supported, and expected. Therefore, hypothesis H2 is put forward.

*H2*: Creative opportunity has a moderating role in the creative behavior of virtual idol fans. Perceived cost negatively moderates interest motivation, achievement motivation, social motivation, and profit motivation about the innovative behavior of virtual idol fans. The community atmosphere positively mediates the creative behavior of virtual idol fans in terms of interest, achievement, social, and benefit motivation.

In the creation practice of virtual idols and fans, fans’ hand-painted creation, video creation, song creation and dance creation of virtual idols all need specific professional knowledge, skills and creative support. Therefore, this study introduces the concept of “knowledge and skills” into the study of fans’ creative behavior, regards the innovative knowledge, creative skills, and creativity mastered by fans of virtual idols as fans’ knowledge and skills, and holds that fans’ assessment of knowledge and skills before participating in the creation of virtual idols significantly affects their final creative behavior, thus putting forward hypothesis H3.

*H3*: Creative ability refers explicitly to the positive moderating effect of knowledge skills in interest motivation, achievement motivation, social motivation, and benefit motivation with the creative behavior of virtual idol fans.

## 3 Materials and methods

### 3.1 Research methodology

Based on the quantitative research method of social science, this study aimed to comprehensively and deeply understand virtual idol fans’ creative behavior and psychological processes. Therefore, the research mainly adopted the questionnaire survey method and the semi-structured interview method as the auxiliary method (Creswell and Clark, 2017).

#### 3.1.1 Semi-structured interview

At the beginning of the research, to obtain a comprehensive understanding of the creative motivation, creative situation, creative form, and other issues of the virtual idol fan creators group, a semi-structured interview method was adopted to conduct exploratory research in advance, and some representative fan creators were selected to conduct one-on-one interviews. Based on the literature study, interview questions were designed with the research purpose, the characteristics of fan creation presented in the virtual idol industry, the creative motivation of fan creators, and the influencing factors in the level of creative opportunity and creative ability were explored. At the same time, the interview results were also used to assist in the design of follow-up questionnaire items.

#### 3.1.2 Questionnaire survey method

The questionnaire survey method, which could quickly and effectively collect data and quantify research results, was widely used in social investigation. Therefore, this study adopted the questionnaire survey method, combining the existing mature scale of relevant variables with the research context and interview results to form a questionnaire, and conducted network research through the Questionnaire Star platform. The questionnaire survey aimed to test the accuracy and universality of the hypotheses deduced above and found the rules of fans’ creative behavior.

#### 3.1.3 Statistical analysis

Based on the data collected from the questionnaire survey, SPSS was used to analyze the data of the valid questionnaire and test the hypotheses. The following statistical analysis methods were mainly used:

- Cronbach’s Alpha coefficient was used to analyze the reliability and validity of the scale. The structural validity of the sample data was tested by confirmatory factor analysis.
- Descriptive statistical analysis. This method was used to analyze the relevant variables affecting virtual idol fans’ creative behavior and the samples’ demographic variables.
- *T*-test was used to analyze and compare the differences between fans of different genders and identities (students and social figures) in multiple dimensions of the motivation to create virtual idols.
- Multiple regression analysis and moderating effect tests were used to verify the research hypotheses and answer research questions.

### 3.2 Methodology

This study was based on a quantitative social science research method, aiming to gain a comprehensive and in-depth understanding of the creative behavior of virtual idol fans and their inner thoughts from a psychological perspective. Therefore, the research was mainly conducted using a questionnaire-based approach and supplemented by a semi-structured interview method.

At the beginning of the study, to gain a comprehensive understanding of the creative motivations, creative situations, and creative forms of virtual idol fan creators, the semi-structured interview method was used to conduct an exploratory study, in which some representative fan creators were selected for interviews. Based on the literature research, the interview questions were designed with the purpose of the study and the characteristics of fan creation presented in the virtual idol industry, focusing on the motivation of fan creators and the factors influencing their creative opportunities and creative abilities. The results of the interviews were also used to support the design of the questions in the follow-up questionnaire (Creswell and Creswell, 2017).

As a widely used social survey method, the questionnaire allowed for quick and effective data collection and quantification of research findings. This study used the questionnaire method, combining established scales of relevant variables with the research context, and interview results to form a questionnaire and conduct online research. The objective of the questionnaire survey was to test the accuracy and generalizability of the hypotheses derived in the previous section and to discover patterns in the creative behavior of fans.

Finally, based on the data collected from the questionnaires, the valid questionnaires were analyzed using SPSS to test the hypotheses using the following statistical analysis methods: reliability and validity analysis, descriptive statistical analysis, t-test, and multiple regression analysis and moderated effects tests, as a means of testing the research hypotheses and answering the research questions.

On the one hand, from a theoretical perspective, this study was based on virtual idol enterprises and their online communities. It took the MOA theory as the research framework, combining the research results of motivation theory and self-determination theory to explore the creative behavior of virtual idol fans from a more comprehensive perspective. In addition, at the level of research methodology, this study conducted an in-depth study of the fan creator groups of virtual idols through interviews and questionnaires, breaking away from the single interview method and text analysis method that has been the primary research path in previous studies of the relationship between idols and fans.

### 3.3 Semi-structured interview design and analysis of interview data

This study adopted a semi-structured interview model to understand the perceptions of virtual idol fan creators about their favorite idols and their creative behavior; and to guide them to recall their past experiences when participating in the creation of virtual idols, including the reasons for and factors influencing their creation. Secondly, from the current mode of creation of virtual idols, the creation environment and conditions of virtual idol production companies and their related platforms, this paper explored the motivation of fans’ creation on the one hand and the opportunities and conditions under which fans were willing to devote their time, energy and money to participate in the creation of virtual idols on their initiative on the other. Finally, based on the interview data, the dimensions of fans’ creative motivation and the factors influencing them at the creative opportunities and abilities level were extracted.

Specifically, in the design of the interview outline, the MOA theoretical model was used as the research framework to divide the interviews into four sections. The first part was to explore the interviewees’ preferences for virtual idols and their participation in the creation of virtual idols; the second part was to investigate the interviewees’ motivation for creation; the third part was to explore the influencing factors affecting the interviewees’ participation in creative behavior; and the fourth part was to count the demographic characteristics of the interviewees. At the same time, the interviewees’ answers were used as an entry point to encourage users to dig deeper into their creative motivations and experiences to gain a comprehensive understanding of fan creators and enrich the depth and breadth of the interview data. The main questions were designed in Table 1.

**Table 1:** Key question design.

Main interview questions
Please briefly describe your favorite virtual idol and discuss your experience of following the star.
Please briefly describe virtual idols, including creation time, creation platform, number of works, creation content and form, etc.
What are the main reasons for your spontaneous involvement in the creation?
What were your biggest expectations when you were involved in the creation? Such as money, emotional expectations, etc., and whether they were met?
What was your biggest takeaway from your involvement in creating a virtual idol?
What was your first opportunity to get involved in the creation?
Have you encountered any difficulties or bottlenecks when you were involved in the creation?
What kind of idols/vibes do you think would motivate you to get involved in creation?
What conditions/abilities do you think would motivate you to get involved in creation?
What do you think of the recent creative solicitation activities sponsored by Henian/Genshin Impact and other officials?
Is there anything else you would like to add or ask me about the discussion just now?

This interview adopted a combination of purposive and heterogeneous sampling methods for the survey respondents. In addition, the anime audience had a higher sense of identification with the virtual world and a higher acceptance of, and willingness to watch, virtual idols (Zhao and Hou, 2018). Therefore, this study focused on finding virtual idol fan creators and followed two principles. Firstly, the selected interviewees should have at least one experience of creating their virtual idols; secondly, the selected interviewees should be diversified as possible to obtain a universal perception dimension and consideration logic. According to the above sampling principles, paid interview recruitment posts for fan creators were posted on platforms such as Lofter, Bilibili, Weibo, and so on to find suitable virtual idol fan creators. In the end, a total of 13 interviewees were selected based on the principle of information saturation.

To make the interview data more structured and systematic, this study followed the following three steps to condense and integrate the interview data. Firstly, the interview data was transformed into a “standardized” text to form a formal text. Secondly, according to the purpose of the study, the textual content of the interviews was categorized. The textual expressions related to fans’ motivation to participate in creating virtual idols and their influencing factors were selected. The main content of the text was extracted to form the conceptualization results. Thirdly, based on the conceptualization results, the relevant dimensional categories that influenced fans’ creative behavior were extracted according to the MOA theoretical research model.

According to the analysis of the interview data, interest motivation was the biggest motivation for fan creators to participate in virtual idol creation, and most interviewees also showed more obvious achievement and social motivation. Although only one interviewee explicitly stated that he wanted to earn money through creation as a career path, the majority of the interviewees also wanted to receive some benefit in return. It expressly said they would be motivated by official or platform “monetary incentive schemes” to participate in creation. Therefore, this study also considered the profit motive as one of the motivations for fans to participate in creative work and included the official/platform incentive scheme in the “community atmosphere” as a moderating factor between the profit motive and creative behavior. It was noteworthy that some respondents indicated that their participation in the creation of virtual idols cost them too much time and money, and they did not achieve the expected rewards, which would reduce the frequency of their subsequent creation; others showed their skills in the creation of virtual idols, which positively contributed to their creative behavior.

### 3.4 Questionnaire design

In terms of measuring the relevant variables, this study mainly used the Likert Seven Scale to assign and measure the relevant variables. The measurement scales referred to mature scales for domestic and foreign-related variables and were adapted based on research questions and previous fan creator interviews to fit the research context.

For the scale design of creative motivation, this study mainly drew on the motivation measurement scales for individual behavior both domestically and internationally. It adapted them appropriately based on the research context. In measuring interest motivation and social motivation, based on the theories from Frederick and Ryan (1993), the ‘Interest Motivation’ and ‘Social Motivation’ scales were adapted, while the Cronbach’s Alpha reliability coefficients of each scale were between 0.81 and 0.91. Unlike general user creative behavior, fans’ creation based on virtual idols was mainly based on their love emotions, manifested as their liking and love for their favorite virtual idols. Therefore, based on the analysis of interview results with fan creators, the item “I am often fascinated by my favorite virtual idol” was added to the measurement of interest dimension; In terms of measuring achievement motivation, the Cronbach’s Alpha reliability coefficient of each scale was approximately 0.85, based on the “intrinsic benefit” scale of Kankanhalli et al. (2005) and the “achievement motivation” scale of Yang et al. (2019); In the measurement of profit motivation, the primary reference was Tang et al. (2012) and Park and Lee (2021) adapted the “Economic Return Motivation” scale, and the Cronbach’s Alpha of each scale was above 0.90 (Table 2).

**Table 2:** Independent variable – initial measurement items and sources of creative motivation.

Dimension	No.	Measurement issues	Source scale
Interest motivation	IM1	I am often fascinated by my favorite virtual idol	Self-drafted
	IM2	My interest lies in participating in virtual idol creation	Frederick and Ryan (1993)
	IM3	I think participating in virtual idol creation can make me feel happy	
	IM4	I participated in the creation of virtual idols to engage in entertainment and leisure activities in addition to work and study	
Achievement motivation	AM1	I participated in virtual idol creation to make more friends with common interests and hobbies	Kankanhalli et al. (2005); Yang et al. (2019)
	AM2	I participate in virtual idol creation to communicate and share with people with common interests and hobbies	
	AM3	I gained a sense of belonging by participating in virtual idol creation	
Social motivation	SM1	I think participating in virtual idol creation can help me gain recognition from others	Frederick and Ryan (1993)
	SM2	I think participating in virtual idol creation can enhance my sense of value	
	SM3	I think participating in virtual idol creation can enhance my sense of achievement	
Profit motivation	PM1	I participated in the creation of virtual idols to receive certain material rewards	Tang et al. (2012); Park and lee (2021)
	PM2	I participated in virtual idol creation to increase the number of fans and gain profit opportunities	
	PM3	I think participating in virtual idol creation can become my livelihood job	

In terms of measuring perceived cost, referring to Leung and Bai (2013) “Social Media Usage” scale, the perceived cost of fans participating in virtual idol creation was divided into three dimensions: time, money, and energy; In measuring the community atmosphere, referring to Fan’s “Website Atmosphere” scale, three items were set to measure fans’ perception of virtual idol communities and fan communities (Table 3).

**Table 3:** Initial measurement questions and sources for the moderating variable-creative opportunity.

Dimension	No.	Measurement issues	Source scale
Perceived cost	PC1	I think it would take too much of my time to be involved in the creation of virtual idols	Leung and Bai (2013)
	PC2	I think it would take too much of my energy to be involved in the creation of a virtual idol	
	PC3	I think it would cost me too much money to be involved in the creation of a virtual idol	
Community atmosphere	CA1	Official/platform/community incentives will motivate me to get involved in virtual idol creation	Fan and Zhang (2015)
	CA2	Official/platform/community incentives will motivate me to participate in virtual idol creation	
	CA3	The success of others in the official/platform/community has motivated me to get involved in the creation of virtual idols	

In measuring creative ability, the main reference was Watson and Hewett (2006) and Leung and Bai (2013) on the “Professional Skills” scale. Based on this scale, adjusted it appropriately according to the creative characteristics of fan creators and set four items (Table 4).

**Table 4:** Moderating variable-initial measure of creative ability question items and sources.

Dimension	No.	Measurement issues	Source scale
Knowledge skills	OR1	I know about virtual idol creation (graphics, animation, audio and video production, etc.)	Watson and Hewett (2006); Leung and Bai (2013)
	OR2	I am familiar with the performance and use of various virtual idol-creation tools	
	OR3	I think I have the creative power to create virtual idols	
	OR4	I think my creations can enrich the image of virtual idols	

In measuring creative behavior, the social media usage scale developed by Cheikh-Ammar and Barki (2016) and the information technology usage behavior scale developed by Davis (1989) was used to measure the creative behavior of virtual idol fans (Table 5).

**Table 5:** Initial measurement questions and sources for the dependent.

Dimension	No.	Measurement issues	Source scale
The act of creation	CB1	I would like to be involved in the creation of my favorite virtual idol	Cheikh-Ammar and Barki (2016); Davis (1989)
	CB2	I plan to participate in the creation of my favorite virtual idol	
	CB3	I will keep participating in the creation of my favorite virtual idols as often as possible	
	CB4	I will increase the frequency of my involvement in the creation of my favorite virtual idols	

## 4 Results

### 4.1 Hypothesis testing of the independent variable – creative motivation

#### 4.1.1 Correlation analysis

From Table 6, the results of the correlation analysis between the independent variables and the dependent variable all showed significance and all had positive values, indicating a positive correlation and allowing for the next step of regression analysis.

**Table 6:** Results of correlation analysis between variables.

Pearson correlation - standard format							
	Average	Standard deviation	1	2	3	4	5
Interest motivation	4.887	1.276	1				
Achievement motivation	4.804	1.252	0.843**	1			
Social motivation	4.768	1.245	0.853**	0.841**	1		
Profit motive	4.269	1.214	0.522**	0.583**	0.599**	1	
The act of creation	4.682	1.223	0.815**	0.805**	0.797**	0.588**	1

*p < 0.05; **p < 0.01.

#### 4.1.2 Multiple linear regression analysis

Table 7 showed that interest motivation, achievement motivation, social motivation, and benefit motivation all significantly positively affected the creative behavior of virtual idol fans.

**Table 7:** Regression analysis of virtual idol fans’ creative motivation on creative behavior.

Results of linear regression analysis (*n* = 317)						
	Non-standardized coefficients		Standardization factor	*t*	*p*	VIF
	*B*	Standard error	Beta			
Constants	0.297	0.156	–	1.898	0.059	–
Interest motivation	0.355	0.060	0.370	5.940	0.000**	4.587
Achievement motivation	0.258	0.060	0.265	4.320	0.000**	4.434
Social motivation	0.175	0.063	0.178	2.797	0.005**	4.810
Profit motive	0.135	0.037	0.134	3.608	0.000**	1.621
*R* ^2^	0.736					
Adjustments to *R*^2^	0.732					
*F*	*F* (4,312) = 217.236, *p* = 0.000					
D-W values	2.006					
Dependent variable: creative behavior						

*p < 0.05; **p < 0.01.

### 4.2 Hypothesis testing of moderating variables

#### 4.2.1 Hypothesis testing of moderating variables

The results showed that the interaction between interest motivation and perceived cost, between social motivation and perceived cost, and between interest motivation and perceived cost interaction with perceived cost reached significant levels in the creative behavior of virtual idol fans. In contrast, the interaction between achievement motivation and perceived cost did not have a considerable effect, as shown in Table 8.

**Table 8:** Tests for moderating effects of perceived costs.

Results of the moderation effect analysis (*n* = 317)				
Model composition	Standard error	*t*	*p*	*β*
Interest motivation	0.036	17.256	0.000**	0.648
Perceived cost	0.036	7.661	0.000**	0.269
Interest motivation * perceived cost	0.020	−2.768	0.006**	−0.090
R^2^/F	0.719/267.316, *p* = 0.000			
Motivation for achievement	0.039	16.321	0.000**	0.647
Perceived cost	0.038	7.174	0.000**	0.264
Motivation to achieve * perceived cost	0.020	−1.807	0.072	−0.062
R^2^/F	0.698/241.051, *p* = 0.000			
Social motivation	0.040	15.884	0.000**	0.640
Perceived cost	0.040	6.189	0.000**	0.241
Social motivation* perceived cost	0.021	−2.753	0.006**	−0.092
R^2^/F	0.678/219.672, *p* = 0.000			
Profit motive	0.051	7.296	0.000**	0.371
Perceived cost	0.052	7.416	0.000**	0.378
Benefit motivation * perceived cost	0.027	−2.163	0.031*	−0.091
R^2^/F	0.456/87.425, *p* = 0.000			
Dependent variable: creative behavior				

*p < 0.05; **p < 0.01.

Further simple slope analysis revealed that the interaction of perceived cost with interest motivation, social motivation, and benefit motivation all had a significant negative effect on creative behavior (Table 9).

**Table 9:** Results of simple slope analysis for perceived cost.

Adjustment variables	Level of adjustment variables	Regression coefficient	Standard error	*t*	*p*	95% CI	
Interest motivation	Average	0.621	0.036	17.256	0.000	0.551	0.692
	High level (+1SD)	0.557	0.050	11.179	0.000	0.459	0.655
	Low level (-1SD)	0.685	0.034	19.911	0.000	0.618	0.753
Social motivation	Average	0.629	0.040	15.884	0.000	0.551	0.706
	High level (+1SD)	0.559	0.053	10.628	0.000	0.456	0.663
	Low level (-1SD)	0.698	0.040	17.344	0.000	0.619	0.776
Profit motive	Average	0.373	0.051	7.296	0.000	0.273	0.474
	High level (+1SD)	0.305	0.059	5.156	0.000	0.189	0.421
	Low level (-1SD)	0.442	0.061	7.206	0.000	0.322	0.562

Each sub-variable under the creative motivation dimension was included in the model as the independent variable, creative behavior as the dependent variable, and community atmosphere as the moderating variable for testing. The effect on virtual idol fans’ creative behavior was substantial. The results showed that the interaction of social motivation with community atmosphere and interest motivation with community atmosphere significantly affected virtual idol fans’ creative behavior. In contrast, the interaction between interest motivation and perceived cost and between achievement motivation and perceived cost did not have a significant effect, as detailed in Table 10.

**Table 10:** Tests for moderating effects of community atmosphere.

Results of the moderation effect analysis (*n* = 317)				
Model composition	Standard error	*t*	*p*	*β*
Interest motivation	0.035	20.627	0.000**	0.746
Community atmosphere	0.027	4.866	0.000**	0.186
Interest motivation* community atmosphere	0.019	−1.276	0.203	−0.047
R^2^/F	0.690/232.091, *p* = 0.000			
Motivation for achievement	0.037	19.518	0.000**	0.737
Community atmosphere	0.028	4.039	0.000**	0.159
Achievement Motivation* community Atmosphere	0.020	−1.471	0.142	−0.055
R^2^/F	0.666/207.762, *p* = 0.000			
Social motivation	0.041	20.013	0.000**	0.831
Community atmosphere	0.030	−0.967	0.334	−0.042
Social motivation* community atmosphere	0.020	2.704	0.007**	0.103
R^2^/F	0.644/188.346, *p* = 0.000			
Profit motive	0.052	10.784	0.000**	0.558
Community atmosphere	0.036	2.462	0.014*	0.126
Interest motivation* community atmosphere	0.023	3.426	0.001**	0.157
R^2^/F	0.389/66.533, *p* = 0.000			
Dependent variable: creative behavior				

*p < 0.05; **p < 0.01.

A specific test was conducted through simple slope analysis, which found that the interaction of social and profit motives in the community atmosphere, significantly positively affected creative behavior (Table 11).

**Table 11:** Results of simple slope analysis for community atmosphere.

Adjustment variables	Level of adjustment variables	Regression coefficient	Standard error	*t*	*p*	95% CI	
Social motivation	Average	0.817	0.041	20.013	0.000	0.737	0.897
	High level (+1SD)	0.911	0.063	14.577	0.000	0.789	1.034
	Low level (-1SD)	0.722	0.043	16.695	0.000	0.637	0.807
Profit motive	Average	0.562	0.052	10.784	0.000	0.460	0.665
	High level (+1SD)	0.702	0.074	9.474	0.000	0.557	0.848
	Low level (-1SD)	0.422	0.057	7.370	0.000	0.310	0.535

#### 4.2.2 Moderating effects of creative ability

The various sub-variables under the creative motivation dimension were included in the model as independent variables, creative behavior as the dependent variable, and knowledge skills as the moderating variable for testing. All four regression analyses showed no interaction effect between creative ability and knowledge skills.

**Figure 2:**
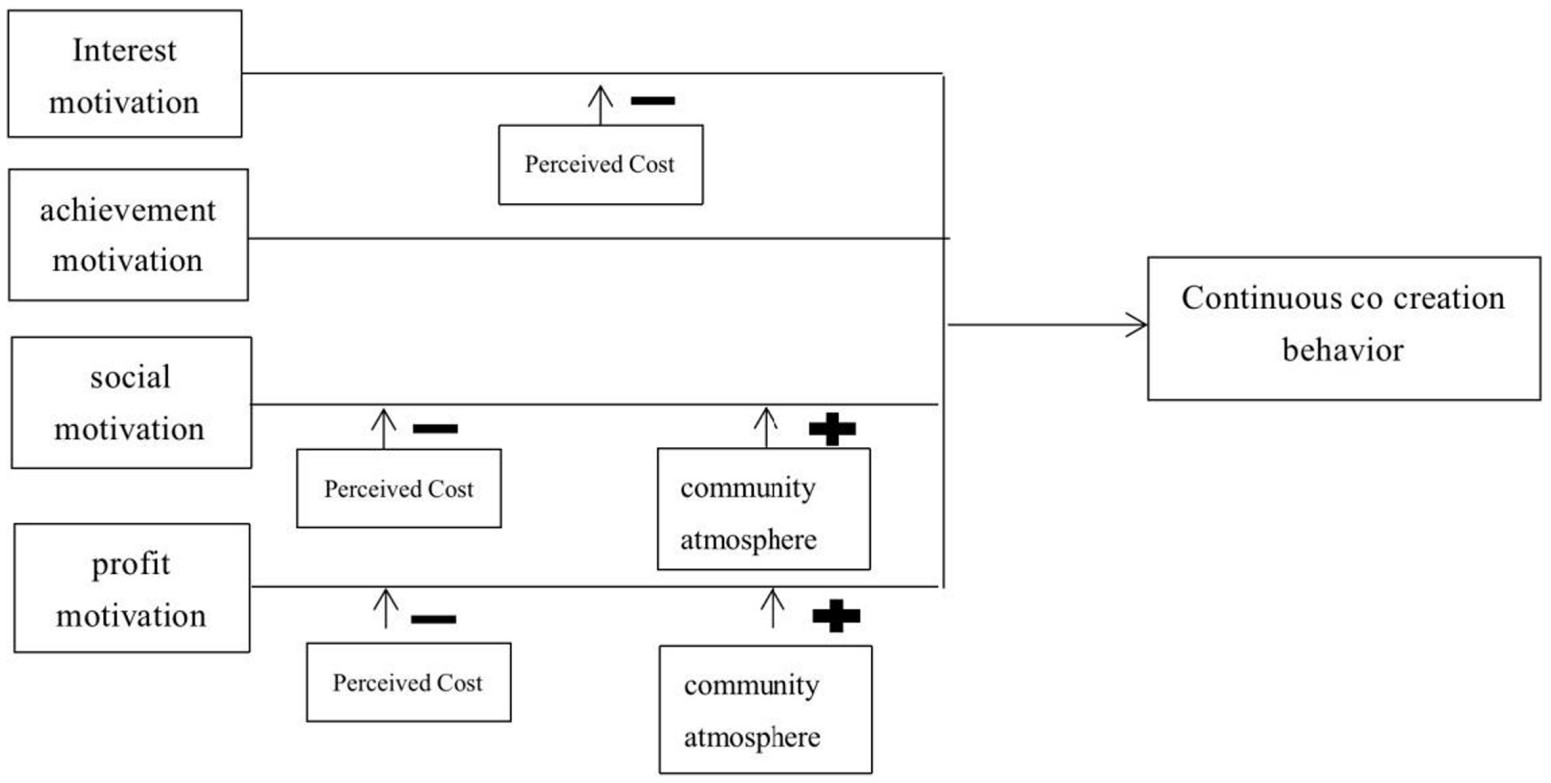
Moderating effects model summary.

Based on the above tests of moderating effects, the following aggregated model of moderating effects could be synthesized (Figure 2).

The valid sample data recovered from the formal questionnaire, and SPSS software was used to conduct reliability tests, descriptive statistics, mean analyses, t-tests, correlation analyses, regression analyses, and moderating effect tests on the sample data to examine the influence of virtual idol fans’ creative motivation on their creative behavior in each dimension and the moderating effects of fans’ creative opportunities and creative abilities in creative motivation. The moderating role of fans’ creative opportunities and creative abilities in their creative motivation and behavior was examined. Based on the results of the data analysis, the hypotheses presented in the previous paper were validated and summarized. In contrast, the graphs of the moderating effect models were summarized for further discussion of the findings.

## 5 Conclusions and discussion

Using the MOA theoretical model as a research framework, this study aimed to analyze the factors influencing the content creative behavior of virtual idol fans. Multiple analysis hierarchical regression analysis was used to test the data. It found that the results were satisfactory and could reveal to some extent, the creative behavior of virtual idol fans in real-life situations. The study was translated by the motivation of virtual idol fans to develop, and the role of external factors of opportunity and ability in moderating this process.

Firstly, this paper concluded that creative motivation had a significant positive effect on the creative behavior of virtual idol fans. Combining motivation theory and self-determination theory, this study classified individuals’ behaviors into four dimensions: interest motivation, achievement motivation, social motivation, and benefit motivation. In the hypothesis testing, the results of the multiple regression model showed that virtual idol fans’ interest motivation, achievement motivation, social motivation, and interest motivation all have a significant positive influence on their creative behavior. In addition, the four dimensions of creative motivation ranked virtual idol fans’ motivation as follows: interest motivation > achievement motivation > social motivation > benefit motivation. Specifically, internal motivation was much more powerful than external interest motivation, and interest motivation was the most critical factor driving fans’ creative behavior. This was also consistent with the results of the previous interviews with virtual idols, i.e., the constant productivity of fans stems mainly from their obsessions and passions. Fan creators primarily processed out of interest and engaged in virtual idol creation primarily out of interest and derived pleasure. Achievement motivation was second only to interest motivation in driving fan creative behavior. Fan creators not only gained pleasure from the process of creation but also achieved satisfaction and added value to themselves. The creative behavior of virtual idol fans was not only a form of entertainment, but also a cultural phenomenon and a way of socializing, and fans were also influenced by their social motivations. In the previous interviews, it was also found that some fans were inspired by their community members to start participating in virtual idol creation; others said they made more like-minded friends and fulfilled their personal social needs through their creation. Some even alleviated their loneliness in reality by participating in virtual idol creation. It was for this reason that the understanding and encouragement of the members of a fan community would not only be one of the driving forces behind virtual idol fans’ engagement in creation but also strengthen the fans’ sense of intimacy and identity with the group in turn, driving them to participate in creation. It is worth noting that the innovative behavior of virtual idol fans is influenced by intrinsic motivations and, to some extent, external interest motivations. In participating in creation, if fans did not receive the expected benefits in return, their intention to participate was weakened. Other fans said that without an official incentive scheme, they might not be motivated to participate in creative work anymore, or they might no longer be encouraged to participate.

Second, the moderating effect of creative opportunities on the creative behavior of virtual idol fans showed different effects. On the one hand, the perceived cost had a significant negative moderating effect on interest motivation, social motivation, benefit motivation, and creative behavior, respectively. In other words, the higher the perceived cost of fans, the greater the cost of effort, time, and money needed to participate in virtual idol creation activities, weakening fans’ willingness to participate and their creative behavior. At the same time, the perceived cost of fan creators also changed with time and state of mind. However, perceived cost only negatively moderated interest motivation, social motivation, benefit motivation, and creative behavior, while it did not pass the significance test for achievement motivation. In other words, the negative effect of achievement motivation, which focused more on the internal sense of achievement and self-fulfillment than on the external perceived cost, may be outweighed by the negative effect of perceived cost due to the pursuit of value and identity of virtual idol creation by virtual idol fans. Fans were willing to devote more time and effort to their idols to help them achieve their own goals. Thus, the moderating effect of perceived costs on achievement motivation was relatively small.

On the other hand, the variable of community climate had a positive moderating effect. The results of the hierarchical regression analysis by introducing community atmosphere as a moderating variable for the creation opportunity dimension showed that community atmosphere had a significant positive moderating effect between social motivation, benefit motivation, and creative behavior. Therefore, a good community atmosphere could promote interaction and cooperation among community members, increase social support and identity, provide more resources and opportunities, and encourage creativity among fans. Taking the more mature Vocaloid virtual idol Luo Tianyi in China as an example, its operating company, Shanghai Huanian Technology, created a good community atmosphere and encouraged fans’ creative participation, such as cooperating with the secondary RMB platform Bilibili Program to release the “Vsinger Creation Incentive Program regularly”; on its official website and its virtual idol, the “AI image collection campaign” was published on the official website and the Weibo accounts of virtual idols such as “Yanhe” and “Le Zhengya”; the “Luo Tianyi MMD model creation Contest.” Most creation competitions were based on traffic ranking and fan voting, which motivated fans to participate in creation with material benefits that were good and excellent. They also created a good creative community atmosphere for fans and inspired them to create. However, the interaction effect of community atmosphere between interest motivation, achievement motivation, and their creative behavior was not significant, indicating that fans’ interest motivation and achievement motivation to participate in creative works were relatively little influenced by community atmosphere. Interest motivation and achievement motivation focus more on the intrinsic needs and fulfillment of the individual rather than the external environment and influence of the community. Coupled with the fact that both were also more dependent on individual interests and talents, they were less influenced by the community atmosphere. Therefore, in virtual idol fan communities, community environment moderated different types of motivation and behavioral performance differently.

Finally, moderating the effect of creative ability on the creative behavior of virtual idol fans, a hierarchical regression analysis was conducted to examine the moderating variable of knowledge skills as a dimension of creative ability. Regarding the moderating effect of creative ability on the creative behavior of virtual idol fans, a hierarchical regression analysis was conducted to examine the moderating variable of knowledge skills as a dimension of creative ability. The moderating effect of creative ability on the creative behavior of virtual idol fans, a hierarchical regression analysis was conducted to examine the moderating variable of knowledge skills as a dimension of creative ability. The moderating effect of creative ability on the creative behavior of virtual idol fans, a hierarchical regression analysis was conducted to examine the moderating variable of knowledge skills as a dimension of creative ability. The results showed that the moderating effect of knowledge skills between interest motivation, achievement motivation, social motivation, benefit motivation, and creative behavior was insignificant, i.e., fans’ knowledge skills did not moderate their creative motivation and creative behavior. This was at variance with previous studies on consumers’ knowledge-sharing behavior in travelers (iQIYI, 2019) and users’ contribution behavior in knowledge websites. The reason for this lay in the specificity of the virtual idol fans, who were a typical group expressing their support and love for their idols by creating songs, drawings, and handicrafts. In this process, not all fans had abundant resources, and participating in the creation may take some time and effort, but this did not affect their passion and motivation for creation. It was also found in the interviews that there was greater flexibility in the form of fan participation in virtual idol creation, covering a variety of forms such as homoeroticism, comics, songs, dance, mashup videos, and cosplay. In general, the specificity of virtual idol fan culture and the flexibility of the forms of their participation in creation lead to a less significant moderating effect of knowledge skills between creative motivation and creative behavior.

### 5.1 Academic implications

This study opened up a new path for the study of virtual idols and fans, bringing about a unique perspective on the study of fans’ participatory creation. In terms of MOA theory research, this study took it as the research framework, which combined the self-determination theory and semi-structured interview results and built a research model of the creative behavior of virtual idol fans. It reasonably divided the influencing factors of virtual idol fans’ creative behavior into motivation, ability, and opportunity. Finally, the analysis has been confirmed through a questionnaire survey. This broadened the application field and research object of MOA theory to a certain extent. As far as the object of study was concerned, this study summarizes and tests the comprehensive factors influencing fans’ creative behavior through semi-structured interviews and questionnaire surveys from a psychological perspective. This also enriched the research of virtual idol fans to a certain extent, especially the antecedents of virtual idol fans’ creative behavior.

### 5.2 Managerial implications

Through empirical research, this study outlined the portrait of the creators of virtual idol fans, promoted the creative development of virtual idols and fans on the one hand, and provided suggestions and strategies for standardizing and promoting the creative behavior of fans on the other hand, and finally achieved the purpose of promoting the healthy and sustainable development of the virtual idol industry. From the perspective of fans, by exploring the influencing factors of fans’ creation, this paper explains the phenomenon of fans’ spontaneous participation in the creation of virtual idols, and provides a management basis for the decision-makers and operators behind virtual idols, to promote virtual idol enterprises and related communities to create excellent creator ecology, designed a reasonable incentive mechanism, and improve fans’ sense of participation and experience. At the enterprise level, by mining the influencing factors of fan creation, virtual idol production companies could help them understand the psychology and behavior of fans’ creation, provide them with marketing management inspiration, and effectively stimulate the potential creative behavior of fans. In addition, promoting the creation of fans could not only enrich the image and number of works of virtual idols, but also improve their exposure, attract more potential fans, and promote the virtuous circle of the virtual idol industry. On the social level, exploring the factors that influenced the continuous creation of fans, virtual idol enterprises, and related communities could encourage fans to create quality content. This could meet the growing demand for diversified entertainment and help fan creators solve economic problems through reasonable business models. In addition, for the teenagers who occupied the main body of fans of virtual idols, clarifying the psychology and behavior of their participation in the creation could help to provide more targeted guidance.

### 5.3 Limitations and further research

The study still suffered from shortcomings in terms of the study population, the scope of the study, and its measurement tools. For future research, the scope of the study should be further expanded. On the one hand, the research object could be extended to the current emerging hyper-realistic virtual idols and compared with the virtual idols for a more comprehensively comparative study; on the other hand, the research object could be subdivided to consider in depth whether fan creators have different influences on their creative behavior when facing different favorite objects. In terms of research content, future research could also explore how factors in the environment of the fan creation system interact to influence fans’ creative behavior from a broader perspective.

Therefore, future research can expand the study population, the scope, and its measurement tools. Further empirical research could also be conducted on the outcomes of sustained fan involvement in creating virtual idol IPs, as well as the marketing initiatives and support of virtual idol companies. Secondly, the measurement tools should be further researched and refined to find a more suitable scale for virtual fans’ creative behavior. Finally, the factors influencing fan creative behavior based on broader theoretical foundations will be explored in depth.

## Data availability statement

The original contributions presented in the study are included in the article/supplementary material, further inquiries can be directed to the corresponding author.

## Ethics statement

Ethical review and approval was not required for the study on human participants in accordance with the local legislation and institutional requirements. Written informed consent from the patients/ participants or patients/participants legal guardian/next of kin was not required to participate in this study in accordance with the national legislation and the institutional requirements.

## Author contributions

QW: Conceptualization, Data curation, Supervision, Writing – original draft, Writing – review & editing. SL: Supervision, Writing – original draft, Writing – review & editing. YZ: Data curation, Writing – original draft. LT: Data curation, Writing – review & editing. YW: Data curation, Formal analysis, Investigation, Supervision, Writing – original draft, Writing – review & editing.
